# Wnt proteins regulate acetylcholine receptor clustering in muscle cells

**DOI:** 10.1186/1756-6606-5-7

**Published:** 2012-02-06

**Authors:** Bin Zhang, Chuan Liang, Ryan Bates, Yiming Yin, Wen-Cheng Xiong, Lin Mei

**Affiliations:** 1 Department of Neurology and Institute of Molecular Medicine and Genetics, Georgia Health Sciences University, Augusta, Georgia 30912, USA; 2 School of Life Sciences, Peking University, Beijing 100871, China

**Keywords:** Wnt, AChR clustering, muscle cells, synapse formation, neuromuscular junction

## Abstract

**Background:**

The neuromuscular junction (NMJ) is a cholinergic synapse that rapidly conveys signals from motoneurons to muscle cells and exhibits a high degree of subcellular specialization characteristic of chemical synapses. NMJ formation requires agrin and its coreceptors LRP4 and MuSK. Increasing evidence indicates that Wnt signaling regulates NMJ formation in Drosophila, C. elegans and zebrafish.

**Results:**

In the study we systematically studied the effect of all 19 different Wnts in mammals on acetylcholine receptor (AChR) cluster formation. We identified five Wnts (Wnt9a, Wnt9b, Wnt10b, Wnt11, and Wnt16) that are able to stimulate AChR clustering, of which Wnt9a and Wnt11 are expressed abundantly in developing muscles. Using Wnt9a and Wnt11 as example, we demonstrated that Wnt induction of AChR clusters was dose-dependent and non-additive to that of agrin, suggesting that Wnts may act via similar pathways to induce AChR clusters. We provide evidence that Wnt9a and Wnt11 bind directly to the extracellular domain of MuSK, to induce MuSK dimerization and subsequent tyrosine phosphorylation of the kinase. In addition, Wnt-induced AChR clustering requires LRP4.

**Conclusions:**

These results identify Wnts as new players in AChR cluster formation, which act in a manner that requires both MuSK and LRP4, revealing a novel function of LRP4.

## Background

The neuromuscular junction (NMJ) is a cholinergic synapse that exhibits a high degree of subcellular specialization characteristic of chemical synapses [1,2]. Its formation is regulated by factors from motoneurons. For example, neural agrin binds LRP4, a member of the low-density lipoprotein receptor (LDLR) family, and subsequently activates the tyrosine kinase MuSK [3-7], leading to the clustering of AChR through mediator proteins including cytoskeletal protein α-actinin [2,8]. Interestingly, muscle fiber prepatterning or aneural AChR cluster formation in the advance of innervation requires MuSK and LRP4 but not agrin, whereas nerve-induced AChR clusters require all [5,7,9]. These observations suggest that MuSK may be regulated by agrin-independent, yet unidentified ligand(s).

Wnt is a family of secreted glycoproteins that play a critical role in development [10]. Wnt signals through a receptor complex consisting of Frizzled (Fz) receptor and LRP5/6 [11]. Fz interacts the adapter protein dishevelled (Dvl) to activate intracellular canonical and non-canonical pathways. Recent studies suggest a role of Wnt in synapse formation. In C. elegans, Wnt signaling determines the position of NMJs by inhibiting synaptogenesis [12] whereas in Drosophila, Wnt promotes the NMJ formation [13,14]. On the other hand, synaptic activity may also regulate Wnt protein expression [15]. Intriguingly, the extracellular domain of MuSK contains a cysteine-rich domain (CRD) that is homologous to that in Fz for Wnt binding [16,17]. MuSK also interacts with Dvl, which regulates agrin-induced AChR clustering [18]. MuSK interacts with LRP4, a close relative of LRP5/6 in the LDLR family [3,4,19]. In zebrafish, Wnt11r binds to *unplugged*, the zebrafish MuSK homologue, to guide motor axons [20]. In mammal muscle cells, agrin-induced AChR clustering was enhanced by Wnt3, but reduced by Wnt3a [21,22].

There are 19 different Wnts in human and mice. Whether and which Wnt is sufficient to stimulate AChR clustering in the absence of agrin remains unknown. Here, we studied the effects of 19 Wnts on AChR clustering in muscle cells and identified five Wnts (Wnt9a, Wnt9b, Wnt10b, Wnt11, and Wnt16) that are able to stimulate AChR clustering, independent of agrin. Expression analysis indicated that Wnt9a and Wnt11 are abundantly expressed in developing muscles. Using these two Wnts as example, we investigated mechanisms by which Wnts regulate AChR clustering. Results indicate that Wnts play an important role in AChR clustering, likely by direct binding to MuSK and in a manner dependent on LRP4.

## Results

### Wnts induce AChR clustering in muscle cells

**Figure 1:**
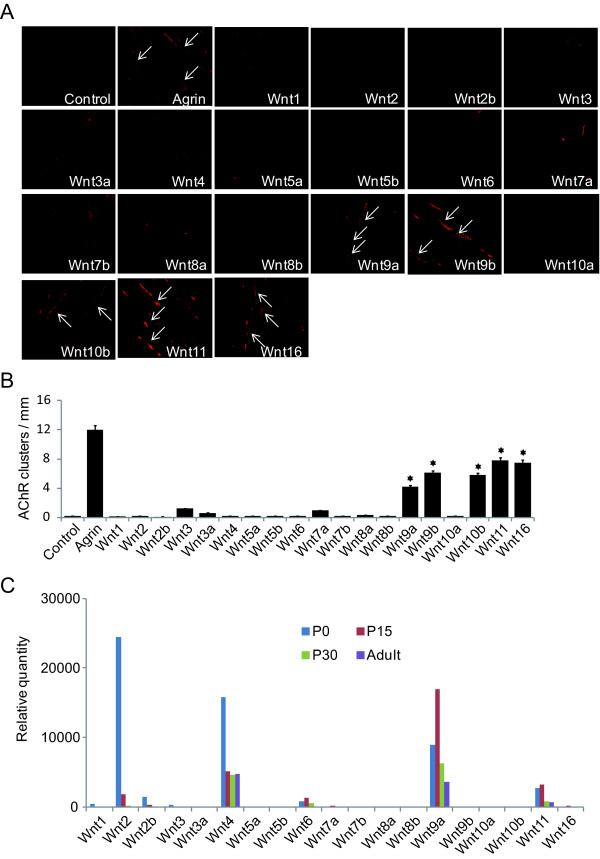
**Wnt proteins induce AChR clusters in muscle cells**. *A*, C2 myotubes were stimulated with conditioned media containing Wnt proteins or control medium for 16 h. AChR clusters were visualized by R-BTX staining and indicated by arrows. *B*. Quantification data of *A*. AChR clusters greater than 4 μm in length were quantified. *, p < 0.01, Student's t test. *C*, Wnt mRNAs are expressed in muscle. Total RNAs were extracted from skeletal muscles of P0, P15, P30 and adult mice and used for qRT-PCR. Relative expression was shown in histograms.

To systematically investigate Wnt function in AChR clustering in mammalian cells, we transfected HEK293 cells with plasmids encoding 19 Wnts (either Flag- or HA-tagged) that have been identified in human and mice. Conditioned media were collected 48 h after transfection. Western blotting by anti-Flag and -HA antibodies recognized the expression of respective Wnts (data not shown). Activity of recombinant Wnts was verified by luciferase activity in HEK293 cells transfected with Top-Flash reporter (data not shown). To study the effect of Wnts on AChR clustering, C2C12 myotubes were stimulated with conditioned media containing a particular Wnt. As control, they were also treated in parallel experiments with conditioned media of non-transfected HEK293 cells or agrin. Sixteen hours after treatment, myotubes were fixed and stained with R-BTX to reveal AChR clusters [3]. As shown in Figure 1A, agrin stimulation increases the number of AChR clusters in C2C12 myotubes, as reported previously [3,18]. Intriguingly, 5 of the 19 Wnts (Wnt9a, Wnt9b, Wnt10b, Wnt11 and Wnt16) were able to induce AChR clusters in the absence of agrin (Figure 1A). This effect appeared to be specific as conditioned medium from non-transfected HEK293 cells had no effect on AChR clustering. These results suggested that Wnt proteins are sufficient to alter AChR clustering in cultured muscle cells. Treatment of C2C12 myotubes by Wnt9a, Wnt9b, Wnt10b, Wnt11 or Wnt16 had no effect on protein levels of AChR or MuSK within 16 h of experiments (data not shown), suggesting that they promote AChR clustering without increasing the levels of AChR or MuSK proteins.

To determine which Wnt(s) may be involved in regulation of NMJ formation in vivo, we examined mRNA levels of 19 Wnts in developing muscles by qRT-PCR. Four Wnts were expressed at levels significantly higher than the rest of the groups: Wnt 2, Wnt4, Wnt9a and Wnt11 (Figure 1C). In this study, we will focus on Wnt9a and Wnt11 because first that they are most abundant Wnts that stimulate AChR clustering as levels of Wnt9b, Wnt10b, and Wnt16 were barely detectable. Second, they are expressed at higher levels at periods most relevant to NMJ formation/maturation.

### Non-additive effect of Wnt and agrin on AChR clustering

**Figure 2:**
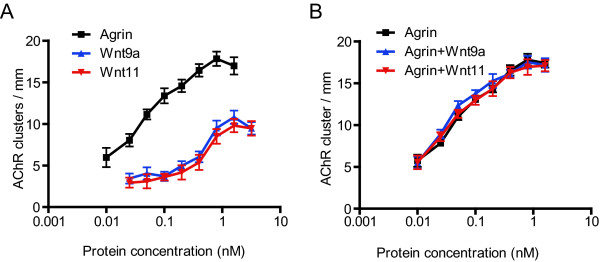
**Characterization of Wnt effect on AChR clustering**. Myotubes were stimulated with increasing concentrations of agrin alone (A) or together with 1 nM Wnt9a or Wnt11 (B). AChR clusters were assayed as in Figure 1.

We next determined if the induction of AChR clusters by Wnt9a and Wnt11 was dose-dependent. Recombinant Wnt9a and Wnt11 were purified from the respective conditioned media by affinity chromatography using Flag-M2 Affinity Gel. As shown in Figure 2A, treatment with increasing concentrations of Wnt9a and Wnt11 led to elevated numbers of AChR clusters in myotubes, indicating that their effects were concentration-dependent. The effects were saturable, with EC_50 _(half maximal effective concentration) values around 0.5 nM, indicating that Wnts act by activating high-affinity receptors. The maximal response of Wnts, however, was about 50% of that for agrin, suggesting that Wnts may not be as efficient as agrin in initiating pathways leading to AChR clustering.

To investigate mechanisms by which Wnts stimulate AChR clustering, we investigated possible functional interaction between Wnt and agrin-initiated pathways. If Wnts and agrin stimulate AChR clusters by different mechanisms, their effects should be additive - the sum of agrin- and Wnt-induced responses. Alternatively, their effect could be synergistic or inhibitory. These are important questions because answers could shed light on potential mechanisms of Wnt-induced AChR clustering. To this end, myotubes were treated with increasing concentrations of agrin together with or without Wnt9a or Wnt11 at 1 nM, concentrations to elicit maximal response. The dose-response curve of agrin in the absence of Wnts superimposed with those in the presence of Wnt (Figure 2B), indicating no change of EC_50 _or the maximal response of agrin by Wnt. These results did not support additive, synergistic, or antagonist effects between agrin and Wnts. Instead, these pharmacological studies suggest that Wnts induce AChR clusters via similar mechanisms of agrin.

### Dependence of Wnt-induced AChR clustering on MuSK

**Figure 3:**
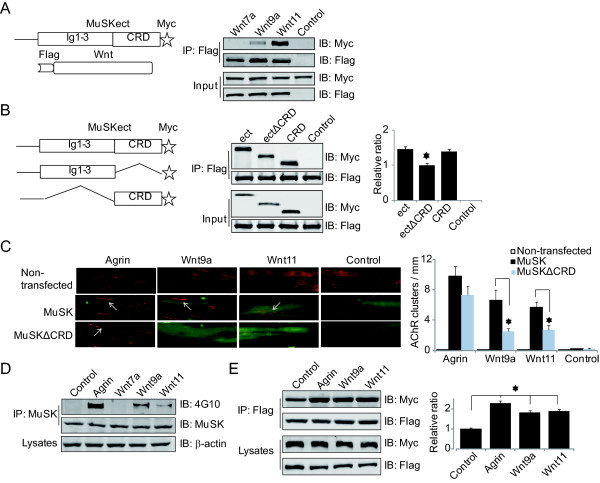
**Wnt effect on AChR clustering requires interaction with MuSK**. *A*, Wnt9a and Wnt11, but not Wnt7a, bind MuSK in vitro. Flag-Wnts immobilized beads were incubated with MuSKect-Myc. Bound proteins were isolated, resolved by SDS-PAGE and blotted with antibodies against Myc and Flag. *B*, Wnt11 binds CRD of MuSK. Flag-Wnt11 immobilized beads were incubated with MuSKect, MuSKectΔCRD or MuSKCRD. Interactions were assayed as in A. *, p < 0.05, n = 3. *C*, Wnt-induced AChR clustering is rescued by MuSK, but not MuSKΔCRD, in MuSK-/- cells. MuSK-/- cells were transfected without or with respective constructs and resulting myotubes (identified by GFP encoded by the expression construct) were assayed for AChR cluster formation in response to agrin or Wnts as in Figure 1. Arrows, AChR clusters. *, p < 0.01, Student's t test. *D*, Wnt9a and Wnt11 induce MuSK tyrosine phosphorylation. C2C12 myotubes were treated by agrin or Wnts for 1 h. MuSK was purified by immunoprecipitation and probed with the 4G10 antibody. *E*, Wnt9a and Wnt11 induce MuSK dimerization. Flag-MuSK and MuSK-Myc were transfected into C2C12 myoblasts and resulting myotubes were treated by Wnts, agrin or control media for 1 h. Flag-MuSK was precipitated by Flag antibody and associated MuSK-Myc was determined by anti-Myc antibody. Band intensity of immunoblot was analyzed by ImageJ software. *, p < 0.01, one-way ANOVA with Student's t test.

MuSK is critical for agrin-induced AChR clustering [5] as well as aneural AChR clustering (or prepatterning) prior to the arrival of motoneuron axon terminals [23,24]. In Zebrafish, Wnt11r was shown to interact with the MuSK homologue *unplugged *and induce AChR prepattern [20]. Having demonstrated that Wnts and agrin may induce AChR clusters via similar mechanisms, we determined if Wnt-induced AChR clustering requires interaction with MuSK. First, we determined if Wnt9a and Wnt11 binds to MuSK directly. Flag-tagged Wnt9a and Wnt11 were immobilized on beads and incubated with MuSKect-Myc, a secreted form of MuSK's extracellular domain fused with Myc. As shown in Figure 3A, MuSKect-Myc was associated with Wnt9a and Wnt11. In contrast, as control, Flag-Wnt7a, which did not stimulate AChR clustering (Figure 1), did not interact with MuSKect-Myc (Figure 3A). These results indicate direct interaction between the two Wnts and MuSK and suggest that Wnt may induce AChR clustering through interaction with MuSK.

Next, we determined which domain in MuSK is necessary for interaction with Wnts. MuSK has a CRD that is homologous to the CRD in Fz [16,17], which is known to bind to Wnts [25]. The CRD in zebrafish *unplugged *is necessary for interaction with Wnt11r [20]. To determine whether the CRD of mammalian MuSK is also necessary for binding to Wnts, we generated ectΔCRD, a mutant MuSKect without the CRD. As shown in Figure 3B, deletion of the CRD significantly attenuated the binding activity, indicating a necessary role of this domain. However, the binding activity was not abolished, suggesting possible involvement of other domains in binding to Wnt. As expected, the CRD alone is sufficient to interact with Wnt (Figure 3B). These results suggest that Wnt interaction by mammalian MuSK involves CRD as well as other domains.

To determine whether MuSK is required for Wnt-induced AChR clustering, we examined Wnt function in MuSK-/- muscle cells. These cells were derived from MuSK-/- mice and are unable to form AChR clusters in response to agrin [26] (Figure 3C, non-transfected). We generate two expression constructs in pIRES-GFP: pMuSK-GFP and pMuSKΔCRD-GFP, which express Flagged MuSK and MuSKΔCRD, respectively, under the control of a CMV promoter and GFP under the control of an IRES (internal ribosome entry site). Expression of wild-type MuSK in MuSK-/- myotubes enabled them to form AChR clusters in response to agrin. In these experiments, GFP was co-expressed and AChR clusters were quantified only in GFP-positive myotubes. Remarkably, Wnt9a and Wnt11 were unable to induce AChR clusters in MuSK-/- myotubes (Figure 3C), indicating the requirement of MuSK in Wnt-induced AChR clustering. This phenotype was rescued by expression of wild-type MuSK in MuSK-/- myotubes (Figure 3C), in support of a critical role of MuSK. Moreover, Wnt-induced AChR clusters were significantly fewer in MuSKΔCRD-transfected MuSK-/- myotubes, compared to those expressing wild-type MuSK. The deletion of CRD had no effect on agrin-induced clusters, indicating that the requirement of the CRD was specific for Wnt. Together these observations suggest that Wnts induce AChR clusters by directly interacting with MuSK.

### Wnt induces MuSK phosphorylation and dimerization

To investigate mechanisms by which Wnts regulate MuSK, we first determined if Wnt stimulation leads to an increase in MuSK phosphorylation in muscle cells. C2C12 myotubes were stimulated by agrin, Wnt9a or Wnt11 for 1 h. Cells were lysed and MuSK was isolated by immunoprecipitation and analyzed for tyrosine phosphorylation with 4G10, a tyrosine phosphorylation-specific antibody [3]. As shown in Figure 3D, treatment of Wnt9a or Wnt11 enhanced the levels of MuSK tyrosine phosphorylation. In contrast, the phosphorylation was not increased in muscle cells treated with Wnt7a, which did not stimulated AChR clusters (Figure 1A). The levels of MuSK phosphorylation by the two Wnts were apparently lower than that by agrin, in correlation with their efficacies of AChR cluster induction, suggesting that MuSK phosphorylation may be a mechanism by which Wnts regulate AChR clusters.

In response to agrin stimulation, MuSK is thought to form homodimers to activate the intracellular kinase domain [2]. However, evidence is lacking that agrin indeed increases MuSK dimerization. To address this issue and to determine whether Wnts facilitate MuSK dimerization, we transfected C2C12 myoblasts with two vectors: Flag-MuSK and MuSK-Myc, which express Flag-tagged (N-terminal) and Myc-tagged (C-terminal) MuSK, respectively. Resulting myotubes were stimulated without (control) or with agrin, Wnt9a, or Wnt11. Flag-MuSK was precipitated from the lysates by anti-Flag antibody and probed for MuSK-Myc with anti-Myc antibody. As shown in Figure 3E, stimulation with Wnts as well as agrin increased the amount of MuSK-Myc, compared to control, suggesting dimerization of MuSK. This result is in support of the model that Wnts interact with MuSK and cause its dimerization and activation.

### Involvement of LRP4 in Wnt-induced AChR clustering

**Figure 4:**
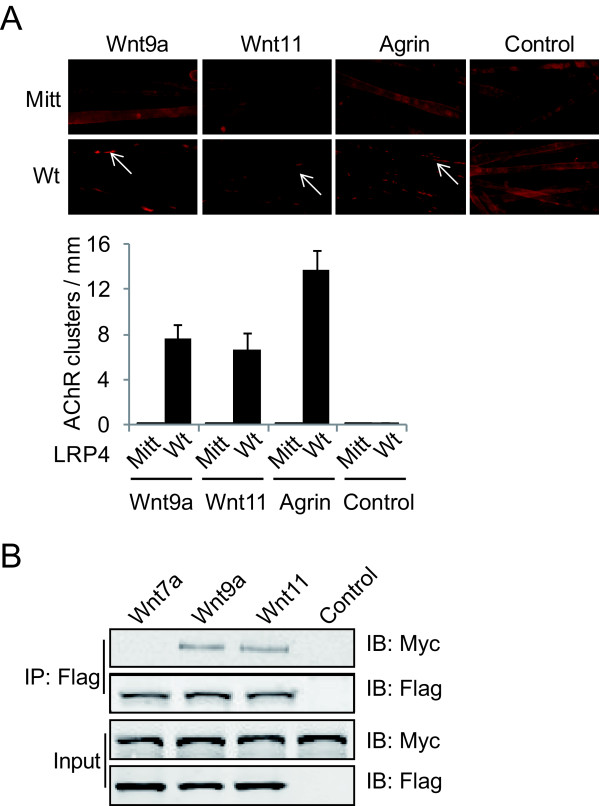
**LRP4 is required for Wnt-induced AChR clusters**. *A*, Wnt9a and Wnt11 fail to stimulate AChR clustering in LRP4^mitt ^muscle cells. Primary muscle cells were cultured from wt and mt mice and assayed for AChR clusters as in Figure 1. Histogram shows quantitative analysis of AChR clusters. *B*, Wnts bind to ectodomain of LRP4. Flag-tagged Wnt7a, Wnt9a or Wnt11 were immobilized with beads and incubated with LRP4N-Myc. Interactions were determined by precipitation and Western blot using anti-Myc antibody.

LRP4 interacts not only with agrin, but also with MuSK, and is critical for agrin-induced AChR clustering and for aneural AChR cluster formation and muscle fiber prepatterning [3,4,9]. We examined whether LRP4 is involved in Wnt-induced AChR clustering in muscle cells. To this end, we cultured primary muscle cells from LRP4^mitt ^null mice. This mutation was induced by ENU, and an early stop codon at the C-terminal to the LDLR type A domains was introduced, which should delete most of the protein [9]. LRP4^mitt ^null mice do not form NMJs. As shown in Figure 4A, agrin stimulated AChR clusters in primary myotubes from wild-type mice, but was unable to do so in myotubes of LRP4^mitt ^littermates, in agreement with previous report [4,9]. Wnt9a and Wnt11 were able to elicit AChR clusters in wild-type primary myotubes, demonstrating that this effect is not limited to clonal C2C12 cells. Interestingly, however, the effect was abolished in myotubes of LRP4^mitt ^null mice, indicating that Wnt-induced AChR clustering requires LRP4, in addition to MuSK.

Next, we determined if Wnt9a and Wnt11 bind to LRP4 using a similar method to study the Wnt-MuSK interaction. Flag-tagged Wnt9a and Wnt11 were immobilized on beads and incubated with LRP4N-Myc, a Myc-tagged recombinant protein containing the extracellular domain of LRP4. As shown in Figure 4B, LRP4N-Myc was associated with Wnt9a and Wnt11. In contrast, as control, Flag-Wnt7a, which did not stimulate AChR clustering (Figure 1A), did not interact with LRP4N-Myc. These results indicate direct interaction between LRP4 and Wnt9a or Wnt11.

## Discussion

In the study we systematically studied the effect of all 19 different Wnts on AChR cluster formation in muscle cells. Wnt9a, Wnt9b, Wnt10b, Wnt11 and Wnt16 were able to induce AChR clustering independent of agrin. In developing muscles, Wnt9a and Wnt11 are expressed relatively abundantly. Using Wnt9a and Wnt11 as example, we demonstrated that Wnt induction of AChR clusters was dose-dependent and non-additive to that of agrin, suggesting that Wnts may act via similar pathways to induce AChR clusters. Wnt9a and Wnt11 function requires both MuSK and LRP4, since Wnt-induced AChR clustering was abolished in MuSK-/- or LRP4 mutant muscle cells. These results identify Wnts as new players in AChR cluster formation.

Wnt regulation of NMJ formation is likely to be complex. In addition to promoting AChR clustering in the absence of agrin, Wnt3 could potentiate whereas Wnt3a inhibits agrin-induced receptor clustering [21,22]. It remains unknown whether LRP4 serves as receptor for Wnts to activate canonical pathways and how these pathways interact with pathways that are initiated by the interaction of agrin and Wnts with the LRP4-MuSK complex. Finally, Wnt signaling is implicated in synapse formation in the CNS. For example, Wnt-7a from granule cells induces axon and growth cone remodeling in mossy fibers [27]. How Wnt regulates CNS synaptogenesis remains unclear. LRP4 is expressed abundantly in the PSD (postsynaptic density) fraction of the brain [28]. It would be of interest to determine whether Wnt regulation of CNS synapse formation requires LRP4.

## Conclusions

This study provides evidence that Wnt9a, Wnt9b, Wnt10b, Wnt11 and Wnt16 are able to stimulate AChR clusters in muscle cells. This effect requires both MuSK and LRP4. Wnt9 and Wnt11 appear to act by inducing MuSK dimerization. These results suggest that Wnts may play a role in NMJ formation.

## Methods

### Reagents and antibodies

Taq DNA polymerase, T4 DNA ligase, and restriction enzymes were purchased from Promega. Horseradish peroxidase conjugated goat anti-mouse and goat anti-rabbit antibodies and enhanced chemifluoresent (ECL) reagents for Western blotting were from Amersham. Rhodamine-conjugated α-bungarotoxin (R-BTX) was from Invitrogen. Oligonucleotides were synthesized by Operon Biotechnologies. Unless otherwise specified, all chemicals were from Sigma-Aldrich. Antibodies were purchased from Sigma (Flag M2, F3165); Upstate Biotechnology (4G10, 05-1050); Novus (β-actin, NB600-501). Rabbit anti-MuSK antibody was described previously [18]. Rabbit anti-LRP4 antibody was described previously [3].

### Constructs

Agrin, MuSK and LRP4 constructs were described previously [3,18]. Expression constructs of Wnt1, Wnt4, Wnt6 and Wnt7b were generously provided by Dr. Xi He, which were fused with an HA tag. To generate Flag-Wnt constructs, Wnt cDNAs (except Wnts described above) were generated by PCR and subcloned into pFlag-CMV1 downstream of an artificial signal peptide sequence and a Flag epitope. Templates for PCR were purchased from Open Biosystems (catalog number in parentheses): Wnt2 (4162686), Wnt2b (8734025), Wnt3 (5726751), Wnt3a (40007188), Wnt5a (3487288), Wnt5b (6438917), Wnt7a (6415801), Wnt8a (40129440), Wnt8b (40056929), Wnt9a (30435371), Wnt9b (5588904), Wnt10a (4921327), Wnt10b (7868324), Wnt11 (40129997) and Wnt16 (40105502). Oligonucleotide sequences for overexpression constructs are omitted due to space limit, but are available upon request. The authenticity of all constructs was verified by DNA sequencing.

### Cell culture and transfection

HEK293 cells and mouse C2C12 muscle cells were maintained as described previously [3,29]. They were transfected with PEI (polyethylenimine, Sigma, 408727), as described previously [30] with modification.

### Recombinant protein production and purification

Agrin was generated and prepared as previously described [18,31]. To produce Wnt recombinant proteins, HEK293 cells were transfected with plasmids encoding Flag- and HA-tagged Wnts. Twenty-four hours after transfection, cells were switched to Dulbecco's Modified Eagle Medium supplemented with 0.05% of fetal bovine serum, and conditioned media were collected and cleaned by centrifugation (1000 rpm, 10 min at room temperature). Control conditioned medium was collected from non-transfected HEK293 cells in parallel experiments. Flag-tagged Wnt7a, Wnt9a and Wnt11 were purified by affinity chromatography using anti-Flag M2 Affinity Gel (Sigma, A2220) per manufacturer's instruction.

### AChR cluster assays

AChR clusters in C2C12 myotubes were measured as described previously with modification [3,29,31].

### Quantitative real time PCR

Total RNA of mouse muscle were extracted using Trizol reagent (Invitrogen) following manufacturer's instruction and transcribed into cDNA templates. The abundance of Wnt mRNA was determined by quantitative real time PCR (qRT-PCR) using appropriate primers and SQBR Green as indicator [32]. GADPH was used as internal control. Oligonucleotide sequences used for qRT-PCR were omitted due to space limit, but are available upon request.

### Solution binding assays and immunoblotting

The solution binding assays and immunoblotting were performed as previously described [3].

### Statistical analysis

Data of multiple groups were analyzed by ANOVA. Two-tailed Student's t test was used to compare data between two groups. Differences were considered significant at *P *< 0.05. Values and error bars in figures denote mean ± SD.

### List of abbreviations

NMJ: neuromuscular junction; AChR: acetylcholine receptor; LDLR: low-density lipoprotein receptor; Fz: Frizzled; Dvl: dishevelled; CRD: cysteine-rich domain; IRES: internal ribosome entry site; PSD: postsynaptic density

## Competing interests

The authors declare that they have no competing interests.

## Authors' contributions

BZ, WCX, and LM conceived the idea, designed experiments, and wrote the paper. BZ executed experiments and analyzed data. CL assisted in AChR clusters assays and YY assisted in generating Wnt constructs. RB assisted in writing the paper. All authors read and approved the manuscript.
